# Delayed Breaker Systems To Remove Residual Polymer Damage in Hydraulically Fractured Reservoirs

**DOI:** 10.1021/acsomega.1c04187

**Published:** 2021-11-16

**Authors:** Bisweswar Ghosh, Mumin Abdelrahim, Debayan Ghosh, Hadi Belhaj

**Affiliations:** † Department of Petroleum Engineering, Khalifa University, Abu Dhabi 2533, United Arab Emirates; ‡ Epygen Labs, Dubai Science Park, Dubai 485018, United Arab Emirates

## Abstract

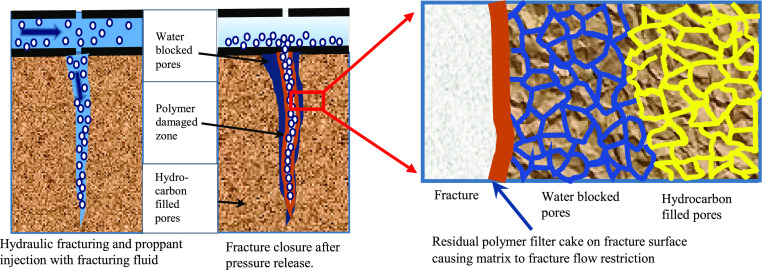

Hydraulic fracturing is a widely used technology to enhance the productivity of low-permeability reservoirs. Fracturing fluids using guar as the rheology builder leaves aside residual polymer layers over the fractured surface, resulting in a restricted matrix to fracture flow, causing reduced well productivity and injectivity. This research developed a specialized enzyme breaker and evaluated its efficiency in breaking linear and cross-linked guar-polymer gel as a function of time, temperature, and breaker concentration targeting a high-temperature carbonate reservoir. The study began with developing a high-temperature stable galacto-mannanase enzyme using the “protein-engineering” approach, followed by the optimization of fracturing fluids and breaker concentrations measuring their rheological properties. The thermal stability of the enzyme breaker vis-à-vis viscosity reduction and the degradation pattern of the linear and cross-linked gel observed from the break tests showed that the enzyme is stable and active up to 120 °C and can reduce viscosity by more than 99%. Further studies conducted using a high-temperature high-pressure HT-HP filter press for the visual inspection of polymer cake quality, filtration loss rates, and cake dissolution efficiency showed that a 6 h enzyme treatment degrades the filter cake by 94–98% compared to 60–70% degradation in 72 h of the natural degradation process. Coreflooding studies, under simulated reservoir conditions, showed the severity of postfracture damage (up to 99%), which could be restored up to 95% on enzyme treatment depending on the treatment protocol and the type of fracturing gel used.

## 1 Introduction

The practice of hydraulic fracturing has found widespread applications in tight and unconventional reservoirs as well as in conventional reservoirs by the virtue of increasing reservoir contact. Making contact with as much reservoir rocks as possible via high-conductivity fracture networks is the primary goal of any hydraulic fracturing job. Thus, the success of a hydraulic fracturing treatment is mainly attributed to appropriate fracture prosperity (mainly the length and width), proppant pack permeability, and flow back capacity.^1^ An ideal fracturing fluid is supposed to have optimum viscoelastic properties for fracture initiation and fracture propagation and the ability to suspend the desired proppant in sufficient concentrations to deliver into the created fractures. It should also degrade sufficiently so that the viscosity of the fracturing fluid allows flow back after the completion of the fracture job and maintains the desired hydrocarbon production.^2,3^ Most hydraulic fracture treatments use an optimum dose of guar-based polymers (guar and its derivatives) as a thickening agent or viscosity builder. Guar gum and its derivatives are known for quick hydration properties, excellent proppant transport, and leak-off control at low concentrations, particularly when cross-linkers are used.^4−6^

Guar gum is extracted from *Cyamopsis tetragonoloba* and made of basic sugars, galactose, and mannose in a 1:1.6 to 1:1.8 ratio.^7^ Because of the cost efficiency, technical competency level, and operation friendliness, guar gum or its variants remain the dominant viscosifier. Guar derivatives, with improved properties, most commonly used in fracturing fluids include carboxymethyl guar (CMG), hydroxypropyl guar (HPG), and carboxymethyl hydroxypropyl guar (CMHPG), adding unique and favorable attributes to each one of them.^8^ The guar molecules tend to aggregate in aqueous solutions in the presence of salts mainly due to intermolecular hydrogen bonding, which poses dual problems: (1) the aggregates may clog formation pores and restrict hydrocarbon flow and (2) the polymer loading required to achieve designed rheological properties would be high. To achieve the required rheology at lower polymer concentrations, several cross-linking agents (multivalent ions) have been used, such as Cr^+3^, Al^+3^, Zr^+4^, and, most commonly, B^–3^. Boron in its trivalent form reacts with the hydroxyl group of galactose linkages and ties multiple polymer strands to form bis-diol complex superstructures.^9^ Proppants mixed with fracturing fluids keep the fractures open when the pressure is released, and ideally, the fracturing fluids are supposed to degrade and flow back to the surface.^10^ However, from experience, 30 to 90% of fracturing fluids stay underground in fractures or rock matrixes, reducing the conductivity of the fractures resulting in “formation damage” and, consequently, causing a greater reduction in oil and gas production than envisaged. Polymer invasion into the micropores, increased bound water on the rock pores, and proppant surface coating by polymers are major concerns. Also, because of fluid adsorption, the matrix expands, reducing the pores and pore throat sizes. The damage mechanisms are more complex than previously envisaged. A comparative analysis of damage mechanisms in fractured gas wells resulted in the identification of several causes. The gel residue damage of the proppant pack due to complex non-Newtonian rheology, viscous fingering through the proppant pack, and unbroken fracturing fluid/polymers within the proppant pack are the most common ones.^11^

**Figure 1:**
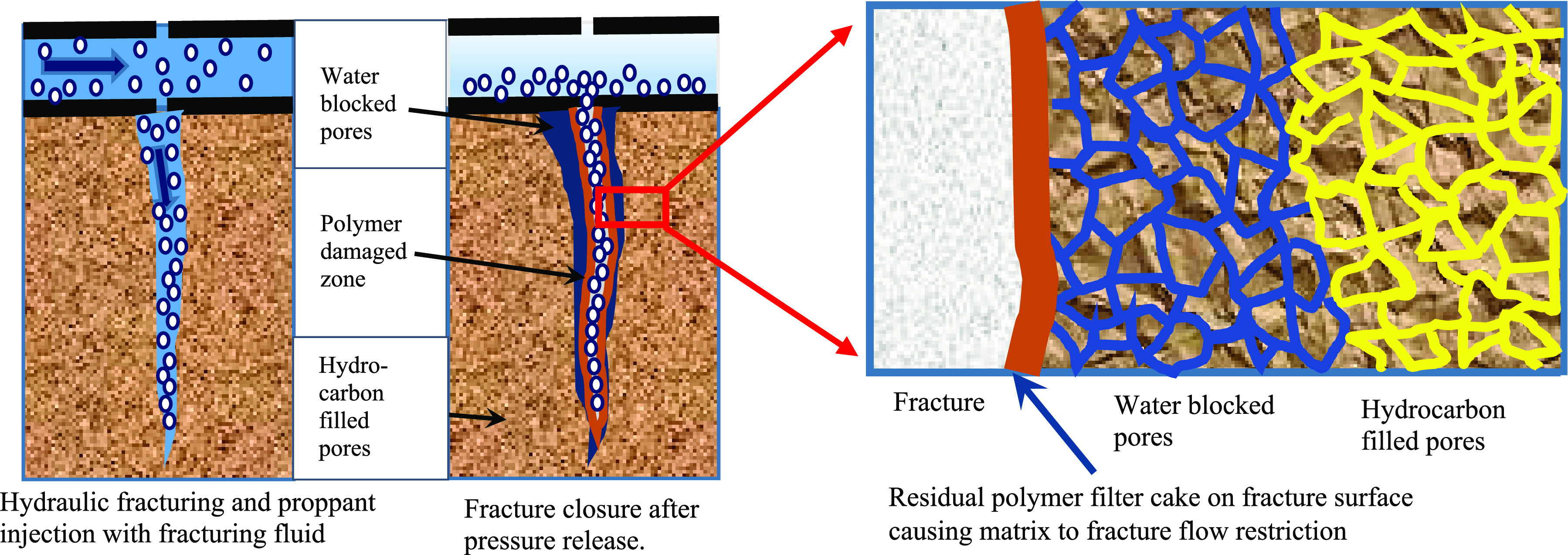
Illustration of a matrix to fracture permeability damage caused by residual polymers and water blockage.

Bose et al.^12^ reported that up to 38% permeability damage on water-sensitive rocks in conventional reservoirs is largely due to the adsorption and retention of polymers and partially because of the bound water around them. Irreversible fracture permeability damage to the extent from 65 to 84% in coal samples has been reported by Huang et al.^13^ on samples from a carbohydrate-binding module (CBM) reservoir. Voneiff et al.^14^ found that unbroken fracturing fluids could lower the overall gas production rate by 30% and reduce initial gas rates by 80% because of the delay in fracture fluid cleanup exceeding weeks or months and suggested the lowering residual fluid viscosity to less than 50 cP. Similar observations were documented from conventional reservoirs through fracture conductivity and core-flow studies in various other laboratories. Because permeability is directly linked to good productivity, this issue poses a significant challenge in realizing the full potential of cost-intensive hydraulic fracturing jobs. Incomplete cleanup due to partial degradation of filter cake and smaller effective fracture lengths because of bypassing of the damaged zone near the tip of the fracture are attributed to the reduction in the conductivity of the hydraulic fractures.^10^Figure 1 shows a simple illustration of the fracture conductivity damage because of the residual filter cake and filtrate invasion into the micropores.

From the above discussion, it is evident that an efficient and specific breaker system that would degrade the adsorbed polymer macromolecules and facilitate complete polymer flowback without incurring additional damage would be welcome by the industry. Oxidizing agents, for example, peroxydisulfates and peroxides, are commonly used as breakers for residue cleanup.^15^ Nevertheless, gradually, the drawbacks of oxidative breakers were recognized and became obsolete. The random and uncontrollable degradation process, limited activities at a lower temperature, and higher threshold concentrations are some of the major drawbacks of oxidizer breakers.^16^ A large amount of residue remaining after the addition of oxidative breakers to the fracturing fluid building strong and impermeable filter cake leads to substantial fracture blockage and reduced hydrocarbon production.^17^ Additional problems with the oxidizers are incompatibility with most organic additives and the special arrangements required during their storage and deployment.^18^ Almubarak et al.^19^ conducted a comparative study with persulfate and bromate as oxidizers and an enzyme (a mixture of 1,6-d-galactosidase and endo-1,4-mannosidase) showing that oxidizers break the polymer gel unevenly, and a significant amount of polymer remained in the solution as clumps. In contrast, the enzyme was able to break the CMHPG evenly and into much smaller fragments. Although oxidizers are relatively common and readily available, their low reactivity and uneven residue removal do not favor their application in fracture jobs. Thus, the interest is shifted to “enzymes” as future potential gel breakers.

**Figure 2:**
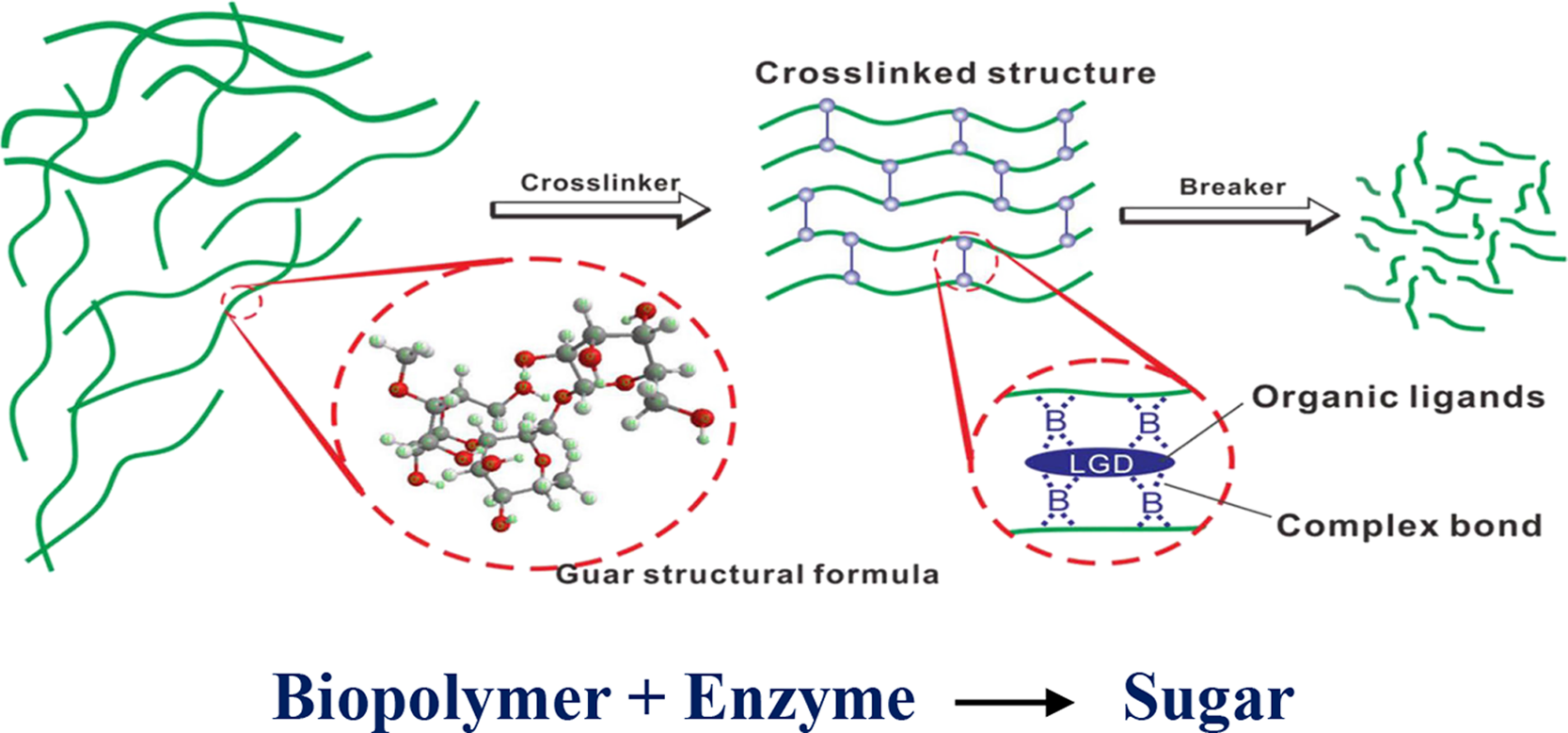
Action of an enzyme on biopolymer macromolecules.

Enzymes are biocatalysts or proteins that effectively catalyze a chemical reaction without being consumed. They are substrate-specific, work under less harsh environments, and are restricted to a specific chemical reaction involving biological molecules.^20^ Enzymes function by a process known as the “lock and key” mechanism, resulting in an enzyme-substrate complex by aligning the matching substrate cleavage at the active enzyme sites. When all sites are aligned, the substrate reacts to form an enzyme-product complex that releases the product, leaving the enzyme unaltered for further attachment to a fresh substrate (causing a superior effect at low concentrations). For enzymatic hydrolysis, the process continues until the substrate is depleted or degraded to the depolymerized simplest form where no further reaction can occur.^21,22^Figure 2 illustrates the polymer breaking mechanism.

Because of the great importance of enzyme activity in attacking the polymer chains, several studies have been conducted on various enzyme breakers that can degrade the glycosidic bonds of guar-based polymers, such as amylose, cellulose, galactose, pectinose, and mannanose.^10,23,24^ There are various types of mannanase enzymes, which act on mannanose polymers (the backbone of guar polymer), but only a certain type exhibits higher activity at high temperatures and a wide pH range.^25,26^ From the comparative studies conducted by Meng et al.^27^ using mannanase, amylase, cellulase, pectinase, and xylanase, it was reported that only mannanase provides a rapid and homogeneous guar-polymer breakage at low concentrations retaining its activity over a wide range of temperatures and pH ranges. However, it was also found that the activity of wild mannanase strain is limited to 150–160 °F. Beyond this range, the enzyme loses its activity.^28^ Most tight gas reservoirs in the Middle East are deep and they have temperatures starting from 200 °F and above. Thus, the need for further development on high-temperature stable and active enzyme breaker is a necessity. Most critically, the enzyme breaker system must be designed in a way such that its activity could be sustained and triggered in a predictable manner, either through thermal activation or chemical activation, or both, so that it does not reduce the gel viscosity prematurely. Zhang et al.^24^ reported the development of a thermostable mannanase enzyme that exhibits a broad spectrum of the activity range (from 80 to 225 °F) and pH up to 10.5, which produced a very low amount of insoluble residues. The authors have also shown pieces of evidence of its superiority over the oxidative breaker (ammonium persulfate) in terms of evenness of breaking, final gel rheology, and fracture conductivity.

This research aimed at developing and evaluating a specialized breaker system that would degrade the adsorbed polymer macromolecules and allow maximum flow back of the residual fracture fluid. It must be suitable for high-temperature applications that do not disturb the viscoelastic properties of the fracturing gel and hence carries the proppants and deliver the necessary fracturing force. For validation and reliability of the development, a coreflood setup and test protocol have been designed to simulate the matrix to fracture flow damage potential and measure the damage removal efficiency of the developed breaker system. To establish the experimental parameter, a target reservoir from the Middle East is selected, whose basic characteristics are given below in Table 1.

**Table 1:** Basic Characteristics of the Target Reservoir

properties	description
location	onshore
vertical depth	11,400–11,580 ft.
formation lithology	tight limestone
formation fracture pressure	8000 psi and above
porosity	15–17%
permeability	10–40 mD
static reservoir temperature	118–120 °C
fracturing fluid temperature while squeezing	90–100 °C

For the fracturing application of borate cross-linked HPG gels, pressure is not a constraint. However, the temperature is of great concern, particularly for deep reservoirs exceeding 320 F (160 °C). There are numerous examples of guar-based gel applications exceeding a wellhead pressure of 6000 psi. In general, the fracturing gel experiences a differential pressure not exceeding 1 psi/ft. while pumping. In our studies, we maintained a differential pressure of 100 psi/ft. or more to maintain the fluid pressure much above its vapor pressure to avoid vapor loss. Higher temperatures affect the stability of guar-based gels as both the glycosidic bonds between the monomer units of the guar and the cross-linking bonds with borate will experience irreversible thermal degradation beyond 320 F.^29^ The degradation process can further accelerate in the presence of oxygen, free radicals, and protons.^30^ If the reservoir temperature is expected to be higher, oxygen scavengers, reducing agents, and pH buffers are used to protect the gel from thermal degradation and enhance the temperature limit.

In the present study, the temperature limit is set at 120 °C not because of the possibilities of thermal degradation of the gel, but it was observed that the efficiency of the gel breaker (enzyme), which is the primary focus of the study, is reduced beyond 125 °C as protein denaturation starts to occur beyond this temperature.

## 2 Materials and Methods

### 2.1 Materials

#### 2.1.1 Fracturing Fluid Ingredients

The guar-based fracturing fluid comprises chemical additives, including a thickener and a cross-linker. HPG guar was used as a thickener and Na-tetraborate as the source of borate ions to cross-link the HPG polymer to further enhance the viscoelastic properties of the fracturing fluid. A high-temperature stabilizer (sodium thiosulfate), pH adjustment chemicals, and all the salts used for preparing brine were sourced from commercial vendors. The linear gel was prepared, and its pH was increased to around 9 by adding 25 wt % of NaOH. Although HPG gel at higher pH produces improved rheology^31^ above pH 9.2–9.3, it is found to precipitate out the divalent ions such as calcium and magnesium present in the formation water when hydroxides are used to control pH.^32^ The cross-linked gel is produced by mixing Na-tetraborate keeping the boron concentration close to 140 ppm. The final pH of the cross-linked gel is also maintained around 9 for the reason mentioned above. The compositions of the fracturing fluids are given in Table 2.

**Table 2:** Composition of Fracturing Fluid

additives	primary function	concentration
HPG guar	viscosifier	3–7 gm/l00 mL
sodium hydroxide (0.1 M)	pH control	as required
acetic acid (0.2 M)	pH control	as required
sodium tetraborate (borax)	cross-linker	50–200 ppm
sodium thiosulfate	high-temperature stabilizer	0.1% w/w

#### 2.1.2 Brines

The formation brine and working brine were synthesized according to the composition of the formation brine obtained from the field laboratory. The fracturing fluid was prepared as per the composition of the working brine available in the field (Table 3).

**Table 3:** Ionic Composition of Formation Brine and Working Brine

ions (mg/L)	Na^+^	Ca^2+^	Mg^2+^	Sr^2+^	Cl^–^	SO_4_^2–^	HCO_3_^–^	ionic strength
formation brine	41,982	10,262	1924	622	88,448	462	337	144,038
working brine	13,004	474	1647	4.4	23,753	3200	134	42,218

#### 2.1.3 Crude Oil

Crude oil was obtained from the target reservoir located in the Middle East. The obtained crude oil is a surface sample that has been degassed and filtered through a 0.3 micron filter to remove any fine particles that may plug the pore throats of the porous media used. The properties of the crude oil are given in Table 4.

**Table 4:** Properties of Crude Oil at Surface Conditions

density at25 °C (g/cc)	viscosity at 25 °C (cP)	total acid number (mg KOH/g)	total basic number (mg KOH/g)
0.8288	5.14	0.34	0.072

#### 2.1.4 Porous Media (Core Plugs) Materials and Preparation

Commercially available Indiana limestone outcrop core plugs were used for the matrix-fracture permeability impairment experiments. Field core samples were avoided as they were too heterogeneous and as it was difficult to find identical samples. The cores were certified to have no clay content, and calcite was the dominant mineral. The plugs were cut, cleaned, and dried until a constant weight was obtained and subsequently saturated with the synthetic formation brine. Porosity and pore volume were calculated using the brine density, and the permeability was calculated by flooding with brine using three separate flow rates. Core properties are listed in Table 5, which reveals very similar petrophysical properties.

**Table 5:** Properties of the Limestone Core Plugs

sample ID	length (cm)	diameter (cm)	He porosity (%)	air permeability (mD)
LsC-1	7.48	3.81	19.67	92.7
LsC-2	7.52	3.80	19.95	86.3
LsC-3	7.51	3.80	20.26	94.2
LsC-4	7.54	3.79	20.15	115.4

#### 2.1.5 Enzyme Breaker

High-temperature, stable, thermally activated enzyme breaker (a robust form of galacto-mannanase) is developed, ensuring minimum denaturation at high-temperature and high-salinity conditions using “directed mutagenesis” and protein-engineering tools and hyperthermophilic bacterium strains. The construction of a mutant library was conducted by the polymerase chain reaction (PCR) and DNA shuffling using the primers. The mutant library containing the pertinent clone was prescreened by adjusting the pH of the plate in the acidic range and applying high-temperature incubation. The clones were further selected by the size of clear hydrolysis halos with measurable diameters. The clones were selected, cultured, and inducted in conical flasks, and subsequently, biochemical assays were conducted to analyze the optimal conditions of growth and expression. The selection of final mutants was done based on the highest biocatalysis at the required pH and temperature conditions. The PCR amplification of the mutant β-mannanase gene was conducted and inserted in the pET 28 plasmid using selected restriction sites and finally expressed in *Escherichia coli* BL 21 cells at various levels of fermentation which ran for 32 h and induced using isopropyl β-d-1-thiogalactopyranoside (IPTG). The purification of protein was conducted by centrifugation, tangential filtration, and ion-exchange chromatography.^33^

Depending on the mechanism of catalyzing the production of oligosaccharides and monosaccharides that can be used for microbial metabolism, mannan-degrading enzymes are classified into different glycosyl hydrolase families (such as GH 1, GH 2, GH 27, and so forth). The primary structure of mannanases in different GH families is different, but they are similar in their spatial arrangement, (β/α)8-barrel protein folds, and are assembled into clan GH-A.^31^ Mannanases often exhibit modular structures consisting of the CBM, catalytic domain(s), and additional functional domain(s).^34^ Under ideal conditions, the enzyme can break down the macromolecular structure into oligomers and monomers attacking specific sites, thus significantly reducing the gel viscosity, enhancing their flowability.

The enzyme was diluted to 2% active volume, and thermal stability was evaluated at 120 °C, using high-pressure/high-temperature (HP/HT) cells, incubated for 4–12 h, showing no denatured coagulates after 12 h of thermal exposure.^35^

### 2.2 Rheology Studies and Gel Optimization

A rheo-pro rheometer was used for these studies. The tests were conducted at temperature intervals from 22 to 80 °C and shear rates up to 100 s^–1^. The studies were conducted for both linear HPG gel and cross-linked gels at different polymer concentrations. Cross-linker concentration and gel pH were optimized and tested against different concentrations of HPG to achieve the highest viscosity and gel stability. Furthermore, investigations on the enzyme activity rates under different conditions were conducted to find the breaking efficiency of the developed enzyme.

### 2.3 HP-HT Filtrate Loss Study

An HP-HT filter press was used to evaluate the fracturing fluid filtration properties and damage characteristics. Measuring filtration properties and observing filtrate and filter cake characteristics is significant to treatment and control. The filter media choice was Whatman cellulose filter papers grade-1, having particle retention ability above that of API standard filter paper and an average pore size of 2.5 μm. The HT-HP filter press mimics filtration against permeable formation at high temperatures and pressures. The optimized fracturing fluid sample was filtered across the filter media, while pressure was applied at the top of the cell and filter loss was recorded. The breaker solution was placed on the top of the filter paper within the HP-HT cell. A pressure differential of 100 psig was applied across the filter paper to create overbalance conditions. The cell was heated to resemble the static reservoir temperature (120 °C). Once filtration loss was measured, the cell was allowed to cool, the filter paper was removed from the bottom of the cell, and the weight of the remaining filter cake was recorded. The filter paper was then placed in an oven at 105 °C for 24 h to dry and measured the dry weight of the filter cake. Three measurements were conducted, and the average was considered as the control.

In repeat tests, the filter cake was allowed to soak for 3 h with the enzyme under the same pressure and temperature. Once cooled, the filter paper was removed, dried, and the dry weight was measured. The cleanup efficiency was measured using the following equation:
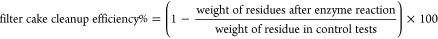


### 2.4 Coreflood Tests To Quantify Fracturing Fluid Damage and Breaker Fluid Efficiency

**Figure 3:**
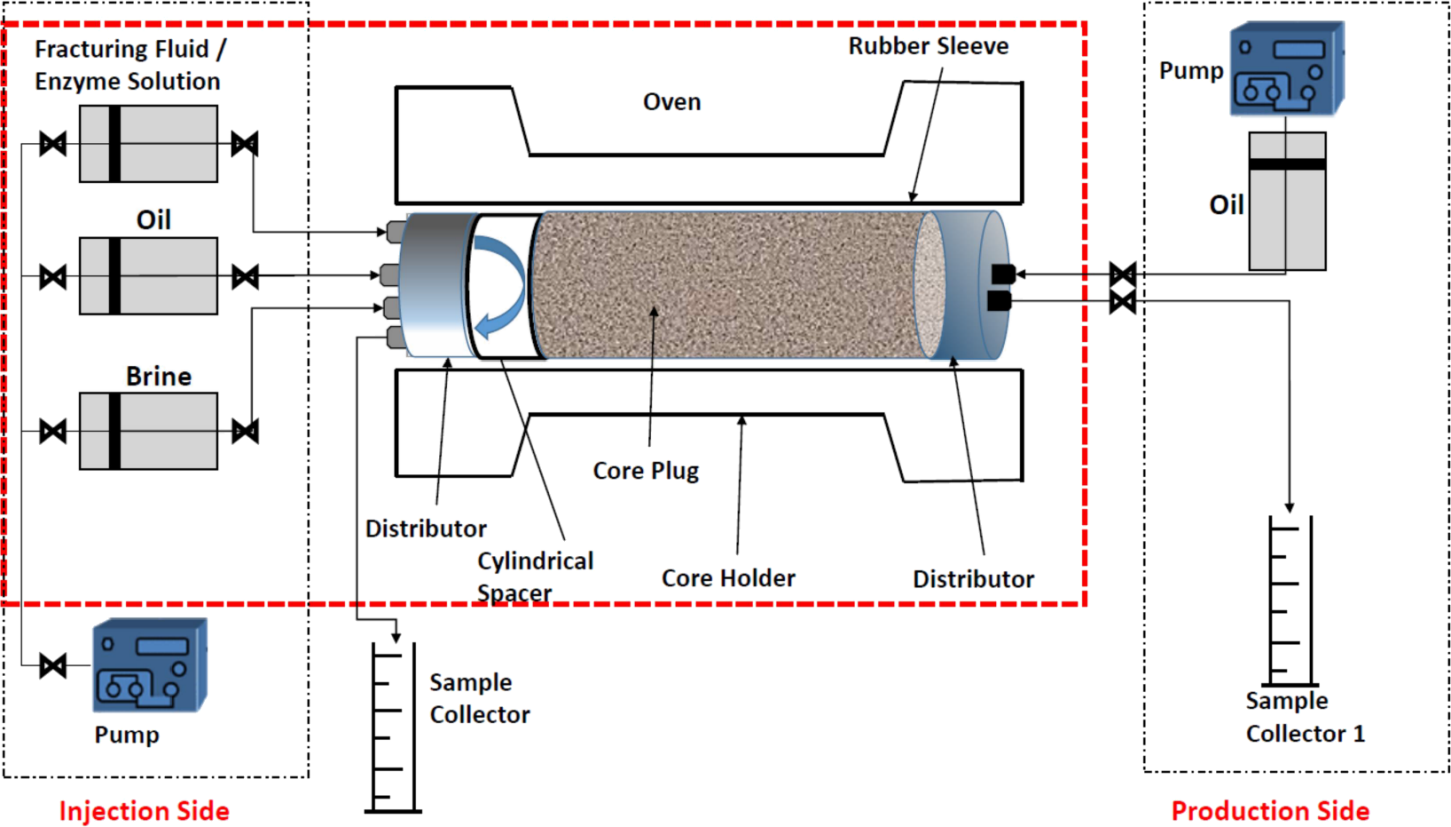
Schematics of the core-flow setup showing the flow direction.

Grace core flooding setup was used for this purpose with required modifications and adjustments so that the fluids can be pumped either in the forward direction to simulate down-hole circulating and injection conditions or from the reverse direction to simulate production through the formation. The core face is designated as the fracture face, and the bulk core represented the matrix. Thus, the setup helped measure the matrix to fracture flow while quantifying the damage. The schematic of the flood setup is presented in Figure 3.

The operating temperature and confining pressure were 120 °C and 700 psig, respectively, with a back pressure of 300 psi. Synthetic reservoir brine was used to assess the initial and residual damages. Four coreflooding tests were conducted using the linear gel, the cross-linked gels as fracturing fluid, and the 2% enzyme solution as breaker fluid. Two different treatment methodologies were applied: first, the fracturing fluid and breaker were injected in sequence, and second, the breaker was mixed with the fracturing fluid, and one-step treatment was conducted (Table 6). Unless mentioned, the fluid flow rate through the cores was maintained at 0.2 mL/min. The stepwise coreflood procedure was as follows:

- Step-1: Measurement of the absolute permeability using formation brine at an injection rate of 0.2 mL/min till the stabilized pressure regime is established.
- Step-2: Establishing *K*_oeff_ at *S*_wirr_ by flowing oil from the production side.
- Step-3: Establishing *K*_weff_ at *S*_or_ by flowing brine from the production side.
- Step-4: Providing static contact between the fracturing fluid and the core face under 100 psi overburden pressure for 4 h or till there was no more filtrate loss. This was performed by keeping the pump at constant pressure mode.
- Step-5: The breaker fluid was circulated at a rate of 3 mL/min for 10 min from the injection side and allowed static exposure with the filter cake for 6 h.
- Step-5: The final step was to determine the postgel breaking return permeability in production mode. Oil and brine were injected from the production direction of the core plug to calculate the production permeability.

**Table 6:** Composition and Cake Removal Efficiencies of Cleanup Solutions

exp no.	details	cumulative filtrate loss
LG-1	linear gel on 3 h exposure	20.1 mL
LG-2	linear gel on 48 h exposure	36.3 mL
LG-3	linear gel on 72 h exposure	41.3 mL
LG-4	linear gel on 6 h exposure with the enzyme breaker	46.2 mL
XG-1	x-linked gel on 3 h exposure	16.3 mL
XG-2	x-linked gel on 48 h exposure	29.7 mL
XG-3	x-linked gel on 72 h exposure	35.2 mL
XG-4	x-linked gel on 6 h exposure with the enzyme breaker	45.9 mL

## 3 Results and Discussion

### 3.1 Gel Optimization and Rheology Investigation

#### 3.1.1 Linear Gel

**Figure 4:**
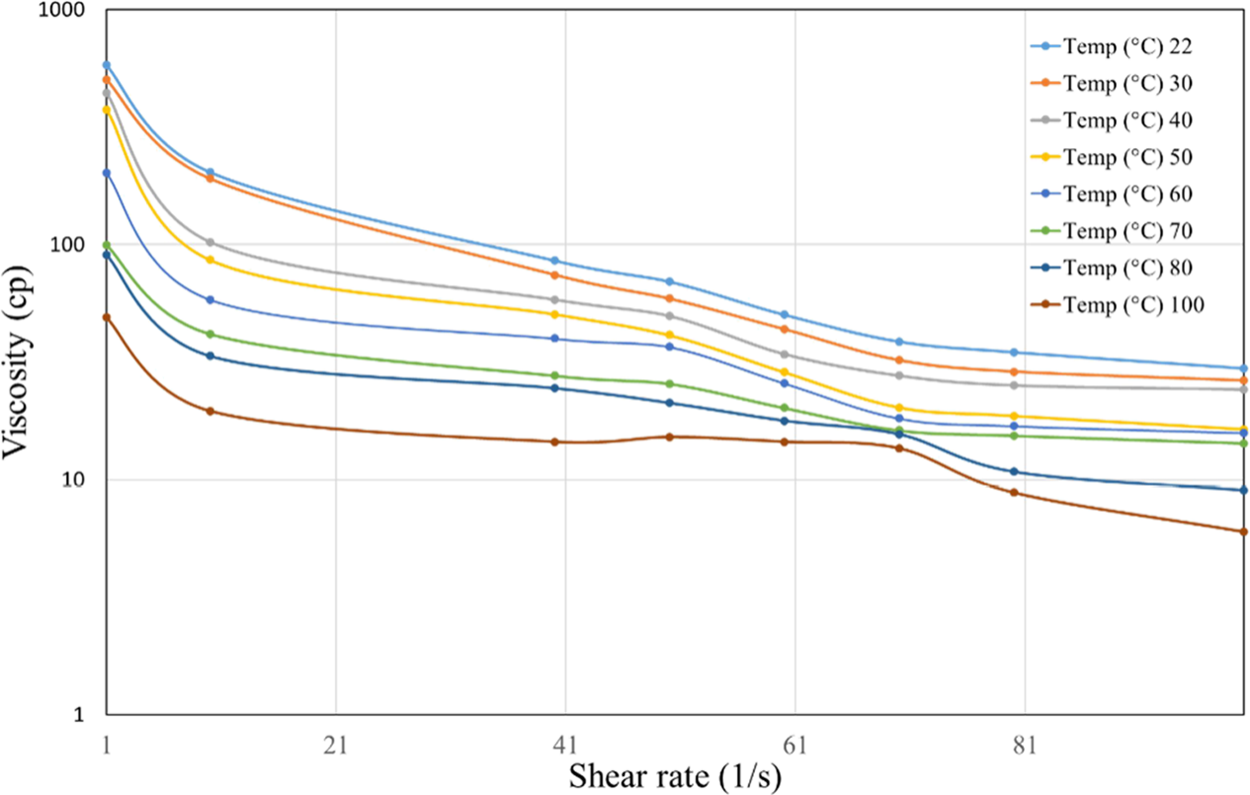
Viscosity vs shear rate of 3000 ppm HPG linear gel at different temperatures.

Experts suggest that the viscosity required to create a fracture for deep and highly consolidated formations and efficient proppant suspension may be in the range of 100–500 cP. However, once the gel reaches the reservoir, the viscosity requirement would be reduced because the temperature effect will be compensated by the reduced shear within the fractures.^36^ Considering the target reservoir parameters given in Table 1 and the coil tubing hydraulics data, the expected maximum temperature and shear rate that the fracturing fluid would experience are 50–70 °C and 100^–s^, respectively, in the coil tube and 90–100 °C and 30–40^–s^ in the fracture.^36^ Considering these figures, the linear gels were prepared with HPG concentrations ranging from 3000 to 7000 ppm for further screening and optimization. Although HPG concentrations were varied in an increment of 500 ppm, because of space constraints, the rheological behavior against temperature and shear rate are presented in log-normal scales for 3000, 5000, and 7000 ppm only (Figures 4^5^,6). From these figures, shear-thinning non-Newtonian pseudoplastic behavior could be seen, and a profound effect of temperature on gel viscosity is also displayed. These results show that to achieve the target viscosity at the coil tube end and the fracture path, 7000 ppm of HPG solution would be optimum.

**Figure 5:**
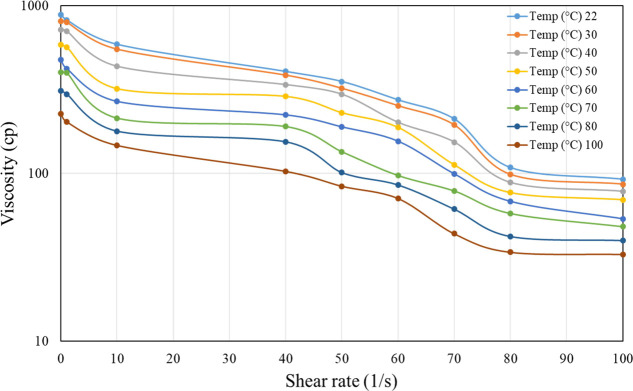
Viscosity vs shear rate of 5000 ppm HPG linear gel at different temperatures.

**Figure 6:**
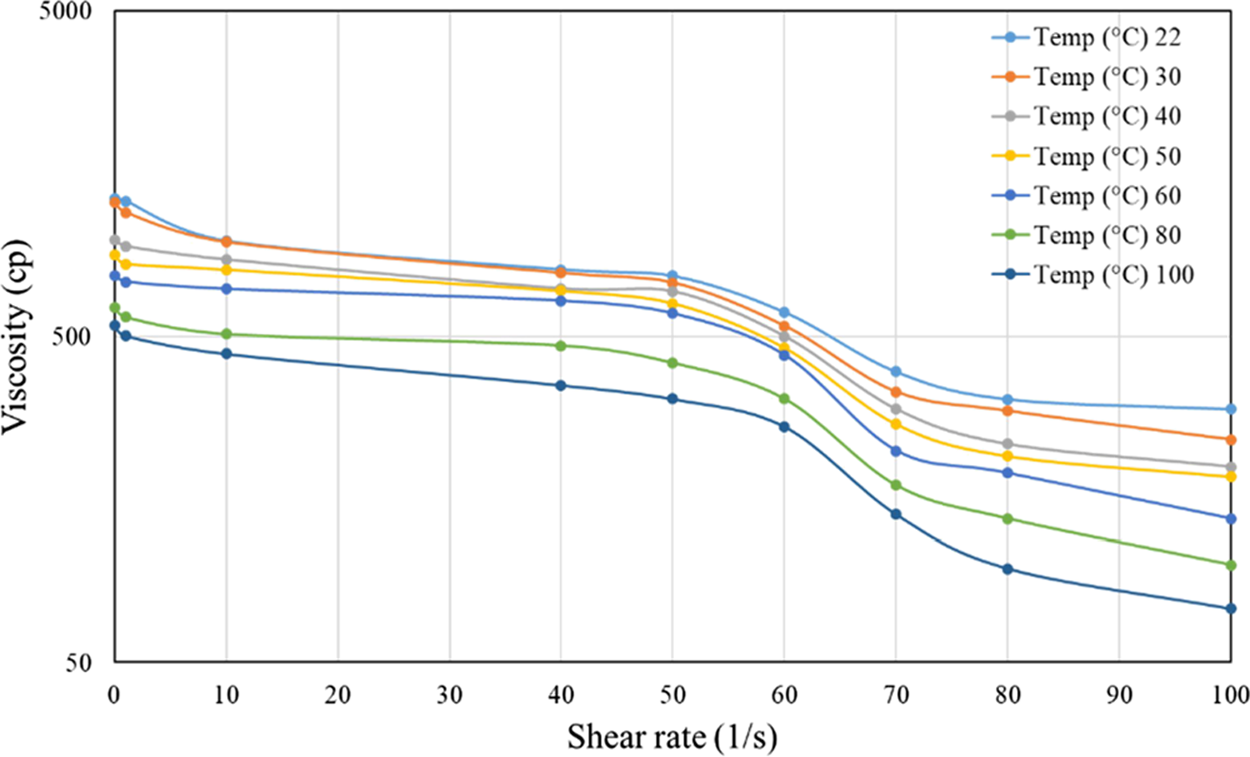
Viscosity vs shear rate of 7000 ppm HPG linear gel at different temperatures.

#### 3.1.2 Cross-Linked Gel

**Figure 7:**
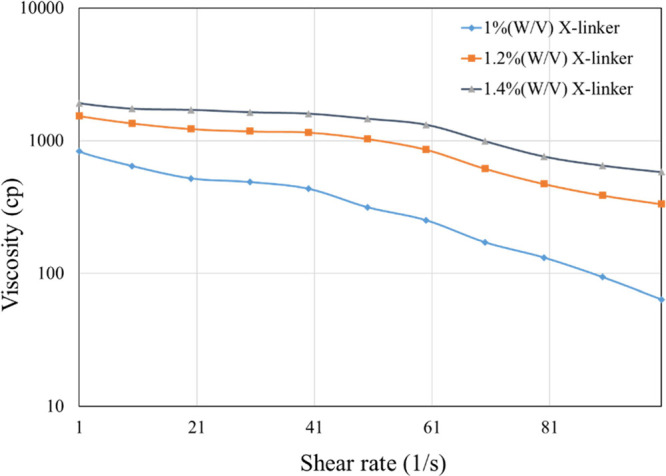
Viscosity vs shear rate of 4000 ppm HPG cross-linked gel at 100 °C with different cross-linker concentrations.

To optimize the cross-linked gel ingredients, the HPG concentration varied from 2000 to 5000 ppm, Na-borate (cross-linker concentration) was varied from 0.8 to 1.2% (W/V) at an increment of 0.2%, and the viscosity was measured at a shear range of 1–100^–S^, at a temperature range from RT to 100 °C. Once again, because of space constraints, only the most accurate data are shown (Figure 7). It is evident that the shear-thinning characteristics are less pronounced in cross-linked gels compared to linear gels. To meet the field requirements, 4000 ppm HPG with 1.2% borate cross-linker was selected as the final composition.

### 3.2 Gel Degradation Studies through the HT-HP Filter Press

The objectives of the HT-HP filter press experiments were to (1) quantify the filtration loss rate for virgin- and breaker-introduced gels, (2) visualize the filter cake quality and thickness, and (3) measure the self-degradation rates and efficiency of enzyme breakers, through the quantification of residual filter cake. Several scenarios were designed, as presented in Table 6, and experiments were conducted in triplicate to minimize measurement errors, and the average values are presented.

Filtrate loss rates were measured with and without enzyme breakers, and the average cumulative filtrate loss for a 60 min measurement period is presented in Table 6. It is evident from these data that the filtration rates of the gel on a nominal exposure of 3, 48, and 72 h aged samples are more for linear gels than against X-linked gels. This can be explained by the fact that water is loosely bound with the polymers in linear gels, and most water molecules are trapped in the cross-linked gel. It is also to be noted that the molecular weight and size of guar molecules are polydispersed (within the range of 0.6–1.6 Da), suggesting the possibilities of a portion of lower size molecules escaping through the filter paper pores. This has resulted in lesser quantities of filter cake in linear gel filtration compared to the cross-linked gel in which the polymer molecules are intricately bound with each other (Table 7). It is also evident from this study that even after 72 h of exposure to reservoir temperature, the cumulative filtrate loss is less than the cumulative filtrate loss when the gels are placed along with the enzyme breaker. In the presence of the breaker, the cumulative filtrate loss at the end of the 60th minute both for the linear and cross-linked gel is very close, indicating faster and more efficient polymer degradation.

**Table 7:** Composition and Cake Removal Efficiencies of Cleanup Solutions

sample name	LG-1	LG-2	LG-3	LG-4	XG-1	XG-2	XG-3	XG-4
initial dry weight (g)	0.3	0.3	0.3	0.3	0.3	0.3	0.3	0.3
wet weight (g)	0.82	0.81	0.8	0.795	0.79	0.82	0.8	0.8
wet weight with cake (g)	6.62	6.22	6.71	6.54	9.1	9.1	9.43	9.49
wet weight after cleanup (g)	4.2	3.5	2.6	0.885	5.3	4.8	3.9	1.4
dry weight after cleanup (g)	1.9	1.1	0.83	0.325	2.9	1.8	1.5	0.465
net weight of the remaining dry filter cake (g)	1.6	0.8	0.53	0.025	2.6	1.5	1.2	0.165
breaking efficiency %	5.88	52.9	68.82	98.52	7.14	46.4	57.14	94.105

**Figure 8:**
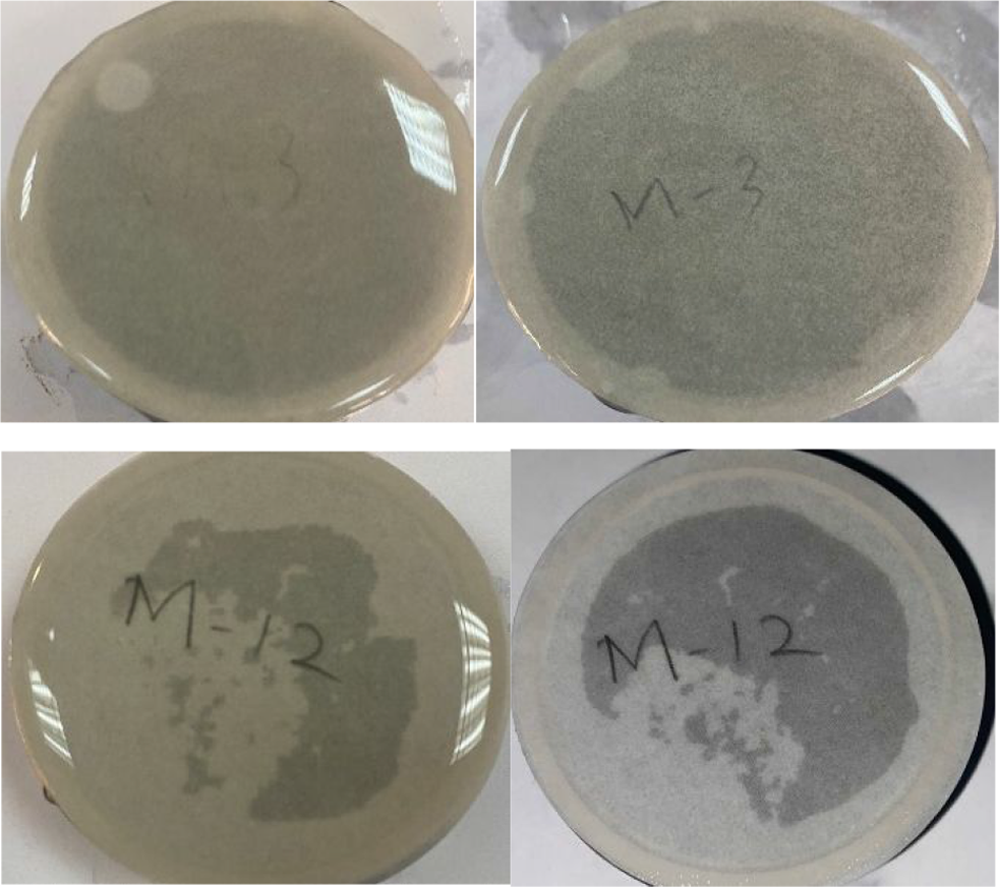
Photographs of filter cakes: Top left: Filter paper with filter cake, Top right: Filter paper after 48 h aging at 120 °C without the breaker. Bottom left: Filter paper with filter cake, Bottom Right: Filter paper after 6 h of enzyme breaker treatment.

To visualize the abovementioned findings, disc sample images are presented in Figure 8. It can be seen from these figures that (1) the overburden pressure exerted on the gel created well-consolidated filter cake, (2) it does not degrade entirely; even after 48 h of thermal exposure at static reservoir temperature (Figure 8 top) and (3), the filter cake treated with the enzyme breaker is almost entirely gone within 6 h of exposure (Figure 8 bottom).

The third objective of this series of tests, that is, quantitative evaluation of residual filter cake, was achieved through the measurement of the dry weight of the filter paper (Table 7). It is evident from this table (correlating with Table 6) that the filter cake-degrading efficiency matches the earlier observations. Both linear and cross-linked gels leave highly damaging polymer residues after 3 h of gel placement, which could be cleaned only up to 69% for liner gels and less than 60% for cross-linked gels even after 72 h of aging at reservoir temperature, indicating the quantum of damage of matrix-fracture flow potential. However, during the enzyme treatment, the damage is removed by 98% for linear gels and 94% for cross-linked gels within only 4 h of exposure. In conclusion, this study proved that the developed enzyme could be a potential guar gel breaker within a short period of time and more effective than the self-degradation process. These encouraging results lead to the coreflood studies at reservoir conditions, which are discussed below.

### 3.3 Coreflood Results and Discussion

**Figure 9:**
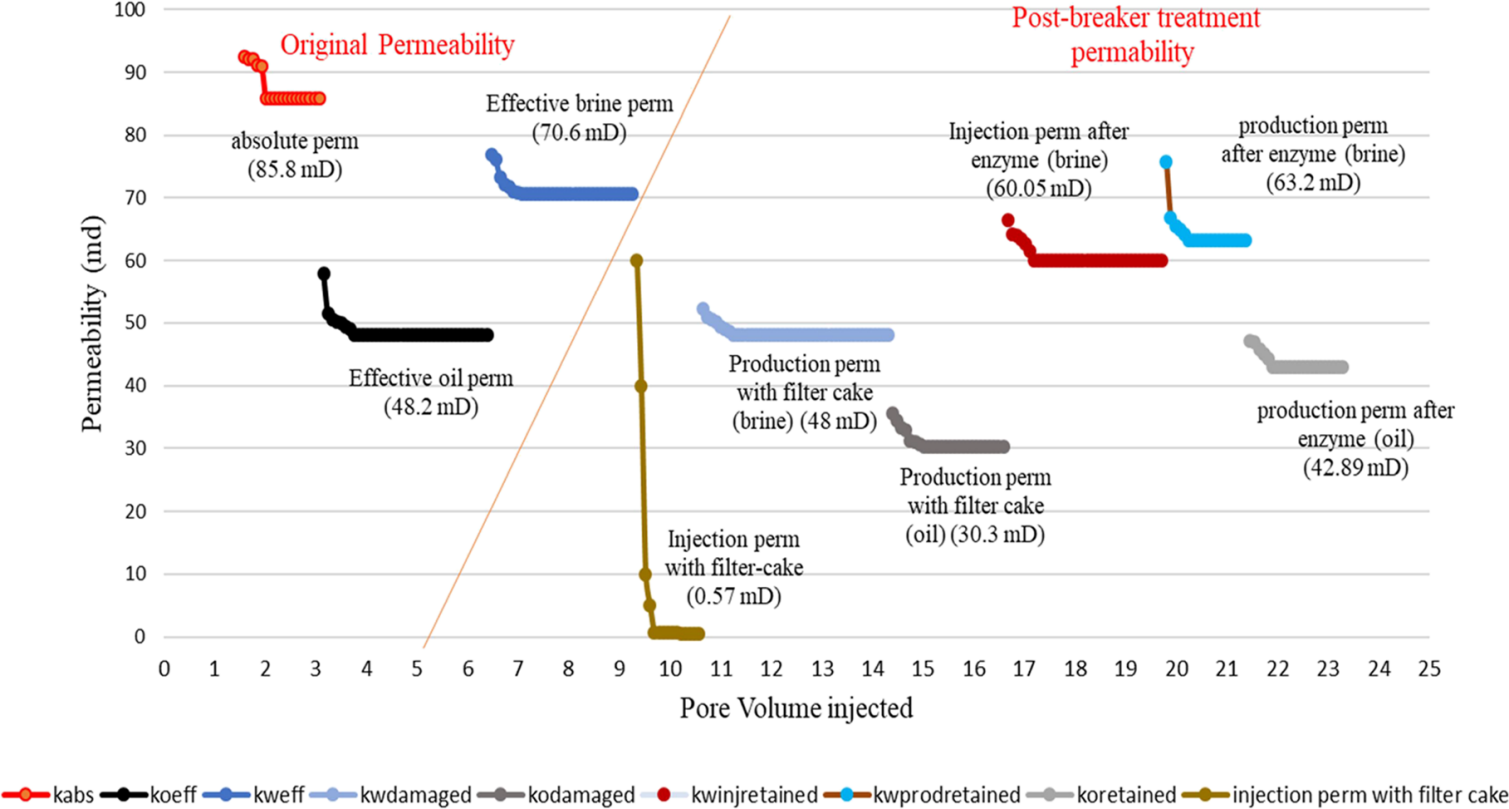
Permeability changes in the case of linear gel filter cake deposition and damage removal using a single-step breaker treatment.

From the above studies, it is understood that a higher proportion of guar gel (both linear and cross-linked) will be depolymerized (self-degraded) with longer exposure to a higher temperature (120 °C in the present case). This supports the observation of Bradley et al.^37^ who reported guar degradation caused by both the increase in heat and flow stress. They also concluded that degradation due to heat is considerably more pronounced than flow-related stress. In the case of fracturing, the fluid is at very low stress once it is placed. Thus, it could be safely assumed that the reservoir temperature is the main factor for polymer degradation. It is also worth mentioning that thermal degradation results in random and heterogeneous degradation, producing polydispersed molecules,^35^ which could be attributed to the presence of a substantial amount of residual filter cake even after 72 h of thermal exposure compared to negligible residues with only 6 h of exposure in the presence of enzymes. To substantiate these observations and to quantify the reservoir flow potential (return permeability) at different damaging conditions, we designed the coreflood protocol by introducing a cylindrical spacer (Figure 3) to represent the wellbore space used for treatment and circulation in actual wells. The main objective of the coreflood study was to investigate the real-time damage of matrix-fracture permeability under reservoir conditions, considering both the hydrocarbon production scenario (matrix to fracture flow) and the water injection scenario (fracture to matrix flow). However, the second and more important objective was to investigate the cleaning efficiency of the developed enzyme breaker and the postfracturing permeability enhancement under the scenarios mentioned above. Four coreflood studies were conducted to meet these objectives, as described in the previous section. The other experimental variables are mentioned in Table 8. Initial core permeability, damaged permeability, and restored permeability because of enzyme treatment are presented graphically in Figures 9^10^,11,12. These results are summarized in Table 9 for the ease of comprehension. On analysis of the coreflood test data, it is seen that the injection permeability of the gel-treated core is reduced by more than 99% (less than 1% of the initial permeability), which is significantly higher than the production permeability damage (37–76%), because of the opportunity to flow back during production. In the case of linear gels with single-step treatment, wherein the enzyme is mixed with the fracturing fluid, the regained injection permeability is above 91% (<9% residual damage), whereas the regained brine and oil production permeabilities are 95.5 and 94.9%, respectively, meaning about 5% permeability damage only. When the treatments were conducted in two steps, that is, the enzyme treatment followed by fracturing fluid treatment, the regained brine injection permeability is seen to be 88.2%, and regained brine, and oil production permeabilities are 93.6 and 91.7% respectively, indicating a slightly reduced effect of the enzyme breaker when placed in sequence. This may be attributed to the inhomogeneous mixing and lesser penetrability of the enzyme into the macrostructure of the gel filter cake.

**Figure 10:**
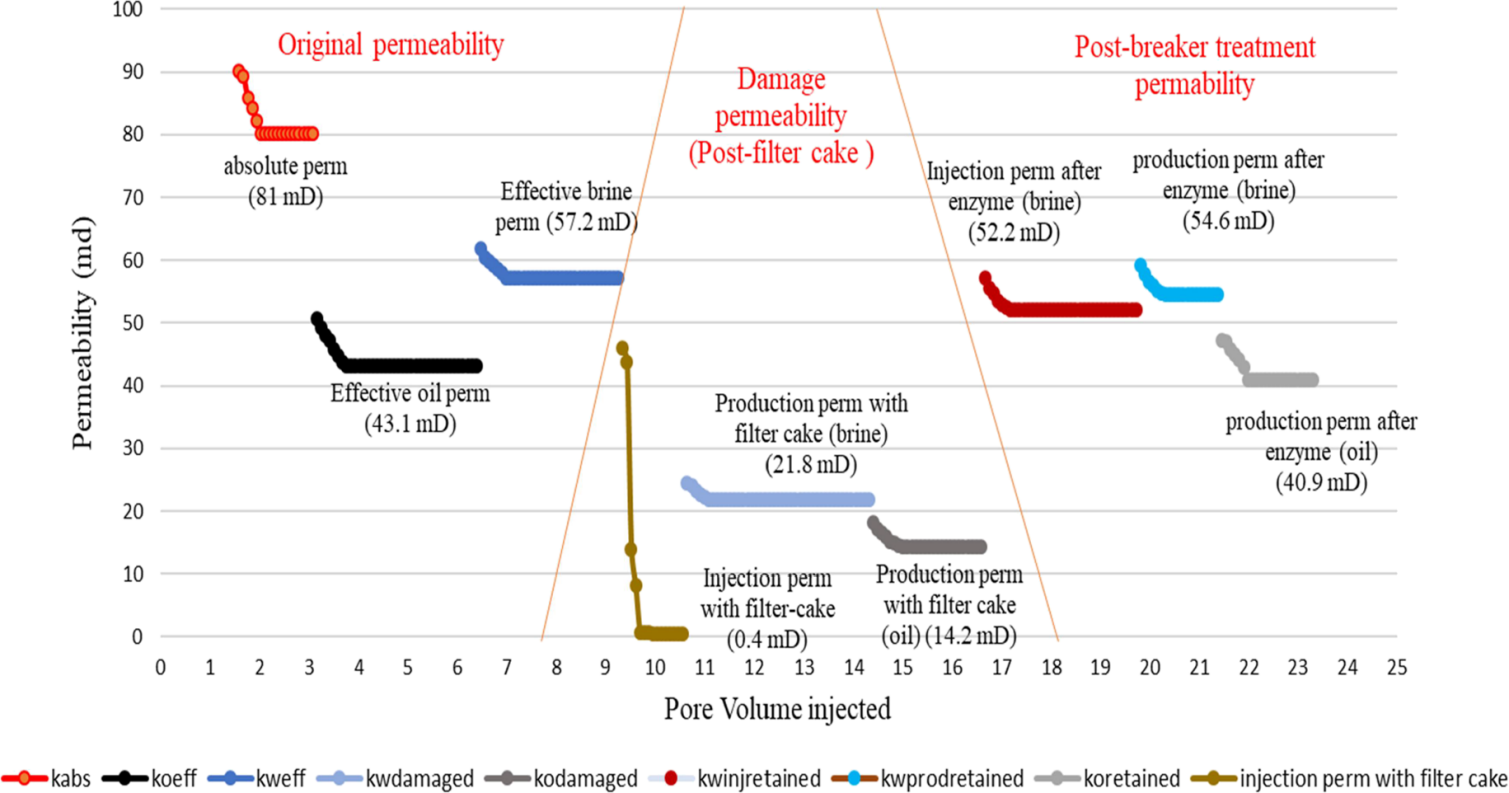
Permeability changes in the case of linear gel filter cake deposition and damage removal using two-step breaker treatment.

**Figure 11:**
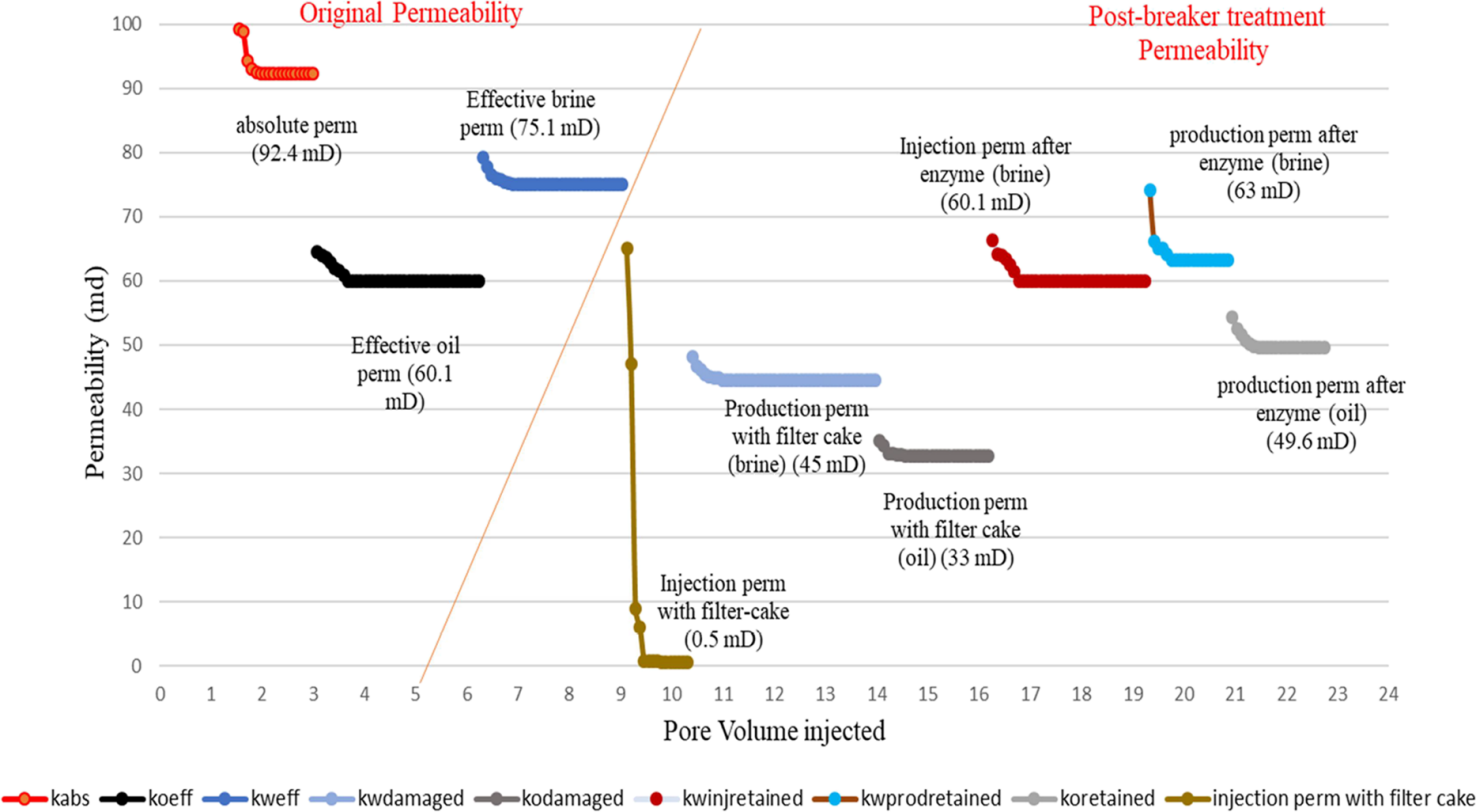
Permeability changes in case of cross-linked gel filter cake deposition and damage removal using a single-step breaker treatment.

**Figure 12:**
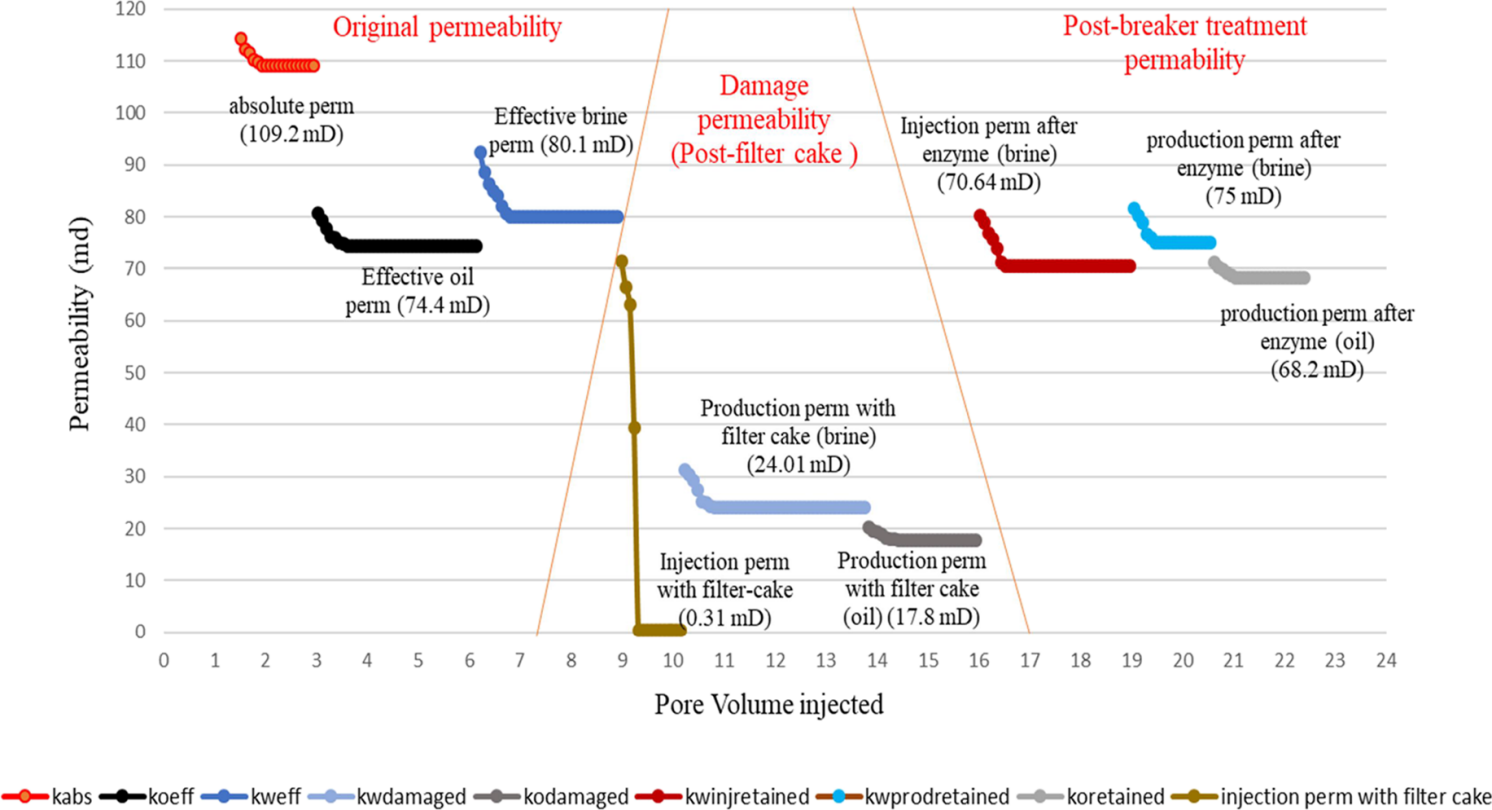
Permeability changes in case of cross-linked gel filter cake deposition and damage removal using two-step breaker treatment.

**Table 8:** Details of Tests with Treatment Fluid Composition

test #	sample ID	description
1	LGCF-1	linear gel mixed with breaker. single-step treatment
2	LGCF-2	linear gel followed by breaker treatment. two-step treatment
3	XGCF-1	cross-linked gel mixed with breaker. single-step treatment
4	XGCF-2	cross-linked gel followed by breaker treatment. two-step treatment

**Table 9:** Coreflood Test Results with Regained Permeabilities

properties	test no./core no.			
	LGCF-1/LsC-2	LGCF-2/LsC-4	XGCF-1/LsC-1	XGCF-2/LsC-3
initial conditions (pretreatment)				
absolute permeability (brine) (mD)	81	109.2	85.8	92.4
effective oil permeability (mD)	43.1	74.4	48.2	60.1
effective brine permeability (mD)	57.2	80.1	70.6	75.1
postfracturing gel treatment				
injection permeability of the core (brine) (mD)	0.4	0.31	0.57	0.5
reduction of injection permeability (brine)	99.5%	99.6%	99.2%	99.3%
production permeability with the filter cake (brine) (mD)	21.8	24.01	48	45
production permeability with the filter cake (oil) (mD)	14.2	17.8	30.3	33
reduction of production permeability (brine)	62%	74.7%	32%	40.1%
reduction of production permeability (oil)	67%	76.1%	37.1%	45%
postenzyme treatment				
injection permeability of the treated core (brine) (mD)	52.2	70.64	60.05	60.1
production permeability of the treated core (brine) (mD)	54.6	75	63.2	63
production permeability of the treated core (oil) (mD)	40.9	68.2	42.89	49.6
regained injection permeability of brine	91.3%	88.2%	85.1%	80%
regained brine production permeability	95.5%	93.6%	89.5%	84%
regained oil production permeability	94.9%	91.7%	89%	82.5%

In the case of the cross-linked gel, the single-step treatment has resulted in 85.1, 89.5, and 89% regained permeability for brine injection permeability, brine production permeability, and oil production permeability, respectively. When the treatments were conducted in sequence (two-step), the brine injection permeability, brine production permeability, and oil production permeability are seen to be 80, 84, and 82.5%, respectively, indicating the reduced effect of the enzyme on sequential treatment.

The coreflood test revealed that the enzyme breaker is more effective in degrading the polymer in the linear form compared to when they are cross-linked. Mannan consists of a backbone of β-1,4-linked mannose residues. Galactose monomers decorate the mannose residues of this hemicellulose through α-1,6 linkages, and the polysaccharides are usually referred to as galactomannans. The hydrolysis of mannose-containing polysaccharides into its monomeric components requires the action of endo-β-1,4-mannanases, exo-β-1,4-mannosidases, and β-1,4-glucosidases.^38^ Boron in its trivalent form reacts with the hydroxyl group of galactose and links multiple polymer strands to form bis-diol complex superstructures.^9^ Once the mannose-containing polysaccharides are broken into smaller molecules, the gel viscosity reduces significantly. However, when the galactomannan assumes a cross-linked macrostructure, the galactose-borate-galactose cross-linking is unaffected by the enzyme reaction and remains mostly intact. This results in less-efficient degradation of the polymer gel into small molecules, and the cleaning efficiency is slightly reduced, as evidenced by higher residual filter cake (seen in HT-HP filter tests) and lesser return permeability observed in coreflood tests. From the coreflood studies, it can be safely concluded that the developed enzyme is a robust breaker system, which can work at temperatures as high as 120 °C, can degrade the damaging polymeric residues on the fracture face, and regain matrix-fracture permeability to the extent of 80–95%, depending on the treatment protocol and the type of fracturing fluid used.

As a way forward, it is proposed to work on a matrix-fracture transmissibility model using the multiple–interacting–continua (MINC) method, in which the coreflood data will be used to derive transmissibility from the MINC proximity function, as described by Ding,^39^ for further evaluating the enzyme activities.

## 4 Conclusions

Parameters from a deep high-temperature reservoir were chosen as the target reservoir, and linear and borate cross-linked fracturing fluid formulation was optimized based on thermal and rheological properties. The prepared fluids were effectively tested and optimized through rheology measurements, and the damage potential was evaluated through HT-HP filtration loss experiments. HPG (7000 ppm) was selected for the linear gel, and 4000 ppm HPG with a 1.2% w/v borate cross-linker was chosen as the cross-linked gel composition along with other ingredients.

A galacto-mannanase-based robust enzyme is developed based on the protein-engineering approach. Its activity and thermal stability were verified. The enzyme concentration was optimized through the rheological studies and HT-HP filter loss method, and it was observed that 2% active concentration has the highest cleaning efficiency, achieving 98% cleanup in HP-HT tests. The major conclusions drawn from this study are as follows:

- Severe matrix-fracture permeability damage can be expected because of the residual gel filter cake on the fracture face, which can go as high as 99% for injection wells and 40–70% for production wells.
- HT-HP filter loss tests revealed that aging the filter cake at the reservoir temperature for as long as 72 h can degrade the filter cake up to 60–70%, whereas the enzyme can degrade 94–98% of the filter cake in 6 h exposure only. The study also shows that linear gels are more prone to thermal and enzymatic degradation than the cross-linked gels.
- It is revealed from the coreflood tests that the injection permeability after fracture could be reduced by more than 99%, whereas production permeability could be reduced by 37–76%. This is quite significant from the good production rate point of view.
- Single-step treatment, wherein the enzyme is mixed with the fracturing fluid; the regained permeability is about 5–7% higher when compared with the two-step treatment, that is, the fracturing fluid treatment followed by the enzyme treatment.
- The data revealed that the enzyme is more effective in degrading the polymer in the linear gel than when they are cross-linked.
- The developed enzyme is a robust breaker system, which works at temperatures as high as 120 °C, degrades the damaging polymeric residues on the fracture face, and regains matrix-fracture permeability to an extent of 80–95%, depending on the treatment protocol and the type of fracturing fluid used.

## References

[ref1] Alshadafan A. H.; Al Kaabi H.; Al Hassani M.; Al Mheiri A. S. Integrated Optimum Design of Hydraulic Fracturing for Tight Hydrocarbon-Bearing Reservoirs. J. Pet. Explor. Prod. Technol. 2020, 10, 3347–3361. 10.1007/s13202-020-00990-6.

[ref2] Rae P.; Lullo G.D. Fracturing Fluids and Breaker Systems. A Review of The State-Of-The-Art. Presented at the SPE Eastern Regional Meeting; Columbus, OH, USA, 1996; Vol. 23–25, pp SPE-37359.

[ref3] Barati R. Nano-Particles as Fluid Loss Additives for Hydraulic Fracturing of Tight and Ultra-Tight Formations. Presented at the International Conference on Ocean, Offshore and Arctic Engineering, OMAE2014, June 8–13, San Francisco, CA, USA, 2014

[ref4] Parris M. D.; MacKay B. A.; Rathke J. W.; Klingler R. J.; Gerald R. E. Influence of Pressure on Boron Crosslinked Polymer Gels. Macromolecules 2008, 41, 8181–8186. 10.1021/ma801187q.

[ref5] Hu Y. T.; Chung H. C.; Maxey J. E. What Is More Important for Proppant Transport, Viscosity or Elasticity?, Presented at the SPE Hydraulic Fracturing Technology Conference, 2015.

[ref6] Ghosh B.; Adi A. S.; Belhaj H. Controlling Excess Water Production in Fractured Carbonate Reservoirs: Chemical Zonal Protection Design. J. Pet. Explora. Prod. Technol. 2020, 10, 1921–1931. 10.1007/s13202-020-00842-3.

[ref7] Al-Muntasheri G. A.; Li L.; Liang F.; Gomaa A. M. Concepts in cleanup of fracturing fluids used in conventional reservoirs: a literature review. SPE Prod. Oper. 2018, 33, 196–213. 10.2118/186112-PA.

[ref8] Hasan A. M. A.; Abdel-Raouf M. E. Applications of Guar Gum and Its Derivatives in Petroleum Industry: A Review. Egypt. J. Pet. 2018, 27, 1043–1050. 10.1016/j.ejpe.2018.03.005.

[ref9] Thombare N.; Jha U.; Mishra S.; Siddiqui M. Z. Borax Cross-Linked Guar Gum Hydrogels as Potential Adsorbents for Water Purification. Carbohydr. Polym. 2017, 168, 274–281. 10.1016/j.carbpol.2017.03.086. 28457450

[ref10] Barati R.; Johnson S.; McCool S.; Green D.; Willhite P.; Liang J. Fracturing Fluid Cleanup by Controlled Release of Enzymes from Polyelectrolyte Complex Nanoparticles. Appl. Poly. Sci. 2011, 121, 1292–1298. 10.1002/app.33343.

[ref11] Friedel T.; Mtchedlishvili G.; Behr A.; Voigt H.D.; Haefner F.K.A. Comparative Analysis of Damage Mechanisms in Fractured Gas Wells, European Formation Damage Conference, 2007.

[ref12] Bose C. C.; Gul A.; Fairchild B.; Jones T.; Barati R. Nano-Proppants for Fracture Conductivity Improvement and Fluid Loss Reduction. Fuel 2019, 251, 30–44.

[ref13] Voneiff G. W.; Robinson B. M.; Holditch S. A. The Effects of Unbroken Fracture Fluid on Gas Well Performance. SPE Prod. Facil. 1996, 11, 223–229. 10.2118/26664-PA.

[ref14] Al-Hulail I.; Al-Khabbaz H.; Al-Janabi Y.; Rahal R.; Dahlan M.; Al-Marshad K. Fracturing Fluid Encapsulated Breaker: High Temperature up to 330 °F, SPE EOR Conference at Oil and Gas West Asia, Muscat, 2018.

[ref15] Soe A.; Azahar B.; Tunio S. Fracturing Fluid (Guar Polymer Gel) Degradation Study by using Oxidative and Enzyme Breaker. Res. J. Appl. Sci., Eng. Technol. 2012, 4, 1667–1671.

[ref16] Sarwar M.; Cawiezel K.; Nasr-El-Din H. Gel Degradation Studies of Oxidative and Enzyme Breakers to Optimize Breaker Type and Concentration for Effective Break Profiles at Low and Medium Temperature Ranges, SPE Hydraulic Fracturing Technology Conference, The Woodlands, TX, USA, 2011.

[ref17] Sumner A. J.; Plata D. L. Oxidative Breakers Can Stimulate Halogenation and Competitive Oxidation in Guar-Gelled Hydraulic Fracturing Fluids. Environ. Sci. Technol. 2019, 53, 8216–8226. 10.1021/acs.est.9b01896. 31276388

[ref18] Almubarak T.; Ng J. H. C.; AlKhaldi M.; Panda S.; Nasr-El-Din H. A. Insights on Potential Formation Damage Mechanisms Associated with the Use of Gel Breakers in Hydraulic Fracturing. Polymers 2020, 12, 2722 10.3390/polym12112722. PMC7698399 33212924

[ref19] Hanssen J.E.; Jiang P.; Pedersen H.H.; Jørgensen J. F. New Enzyme Process for Downhole Cleanup of Reservoir Drilling Fluid Filtercake, SPE International Conference on Oilfield Chemistry, 1999.

[ref20] Sanders M.W.; Twycross J.; Buchan C. R.; Cameron J. J. A. Quantitative Method for Estimating Alfa-Amylase-Based Enzyme Concentrations in Wellsite Field Samples and its Application on a Gravel Pack Completion, American Association of Drilling Engineers (AADE), 2004 Drilling Conference, Houston, 2004.

[ref21] Al-Kitany N.; Ghosh B.; Eldin Y.F.; Al-Bemani A.S.; Al-Hadhrami H. Evaluation of Wellbore Clean up Fluids by Comparing Laboratory Test Results with Field Production Data, In 13th Abu Dhabi International Petroleum Exhibition and Conference (ADIPEC 2008), 2008; pp 1425–1433.

[ref22] Bhatia S.; Singh A.; Batra N.; Singh J. Microbial Production and Biotechnological Applications of Alpha-galactosidase. Int. J. Biol. Macromol. 2020, 150, 1294–1313. 10.1016/j.ijbiomac.2019.10.140. 31747573

[ref23] Zhang B.; Huston A.; Whipple L.; Urbina H.; Barrett K.; Wall M.; Hutchins R.; Andrey M. A Superior, High-Performance Enzyme for Breaking Borate Crosslinked Fracturing Fluids Under Extreme Well Conditions. SPE Prod. Opera. 2013, 28, 210–216. 10.2118/160033-PA.

[ref24] Yin L.; Tai H.; Jiang S. Characterization of Mannanase from a Novel Mannanase-Producing Bacterium. J. Agric. Food Chem. 2012, 60, 6425–6431. 10.1021/jf301944e. 22694324

[ref25] Battistel E.; Bianchi D.; Fornaroli M.; Cobianco S. Enzymes Breakers for Viscosity Enhancing Polymers. J. Pet. Sci. Eng. 2011, 77, 10–17. 10.1016/j.petrol.2011.02.003.

[ref26] Meng Y.; Zhao F.; Jin X.; Feng Y.; Sun G.; Lin J. Performance Evaluation of Enzyme Breaker for Fracturing Applications Under Simulated Reservoir Conditions. Molecules 2021, 26, 31–33. 10.3390/molecules26113133. PMC8197314 34073941

[ref27] Morales R.H.; Gadiyar B.R.; Bowman M.D.; Wallace C.; Norman W.D. Fluid Characterization for Placing an Effective Frac-Pack. SPE Annual Technical Conference and Exhibition; New Orleans, Louisiana, 2001.

[ref28] Shah S.; Asadi M. Fracturing Fluid Characterization: State-Of-The-Art Facility and Advanced Technology; Oklahoma Univ., School Pet. Geolog. Eng., 1997.

[ref29] Chetan P.; Songire S. Sulfur-Free and Biodegradable Gel Stabilizer for High Temperature Fracturing Applications; SPE-175786, SPE North Africa Technical Conference and Exhibition, Cairo, Egypt, 2015.

[ref30] Picout D. R.; Ross-Murphy S. B.; Errington N.; Harding S. E. Pressure Cell Assisted Solution Characterization of Polysaccharides, 2001. Biomacromoleculs , 2 (), 1301–1309. 10.1021/bm010118n 11777407

[ref31] Lia L.; Al-Muntasheri G. A.; Lianga F. A review of crosslinked fracturing fluids prepared with produced water. Petroleum 2016, 2, 313–323. 10.1016/j.petlm.2016.10.001.

[ref32] Srivastava P. K.; Kapoor M. Production, Properties, and Applications of Endo-β-mannanases. Biotechnol. Adv. 2017, 35, 1–19. 10.1016/j.biotechadv.2016.11.001. 27836790

[ref33] Aneesa D.; Kesen M. Applications of Microbial β-Mannanases. Front. Bioeng. Biotechnol. 2020, 8, 1336 10.3389/fbioe.2020.598630. PMC7770148 33384989

[ref34] Al-Taq A. A.; Al-Khaldi M. H.; Alfakher B. M. Successful Application of TSE-Based Fracturing Fluids in Proppant Fracturing for Unconventional Carbonate Source Rock. SPE International Hydraulic Fracturing Technology Conference and Exhibition 2018.

[ref35] Economides M. J.; Nolte K. G. Reservoir Stimulation, 3rd ed.; John Wiley and Sons, 2000.

[ref36] Montgomery C. Fracturing Fluids. Effective and Sustainable Hydraulic Fracturing; 2013.

[ref37] Bradley T. D.; Ball A.; Harding S. E.; Mitchell J. R. Thermal Degradation of Guar Gum. Carbohyd. Polym. 1989, 10, 205–214. 10.1016/0144-8617(89)90012-X.

[ref38] Puls J. Chemistry and Biochemistry of Hemicelluloses: Relationship between Hemicellulose Structure and Enzymes Required for Hydrolysis. Macromol. Symp. 1997, 120, 183–196. 10.1002/masy.19971200119.

[ref39] Ding Y. Modeling of Matrix/Fracture Transfer with Nonuniform-block Distributions in Low-Permeability Fractured Reservoirs. SPE J. 2019, 24, 2653–2670. 10.2118/191811-PA.

